# Editorial: Soilless Cultivation Through an Intensive Crop Production Scheme. Management Strategies, Challenges and Future Directions

**DOI:** 10.3389/fpls.2020.00363

**Published:** 2020-04-03

**Authors:** Nikolaos Tzortzakis, Silvana Nicola, Dimitrios Savvas, Wim Voogt

**Affiliations:** ^1^Department of Agricultural Sciences, Biotechnology and Food Science, Cyprus University of Technology, Limassol, Cyprus; ^2^DISAFA, Department of Agricultural, Forest and Food Sciences, University of Turin, Turin, Italy; ^3^Laboratory of Vegetable Crops, Department of Crop Science, Agricultural University of Athens, Athens, Greece; ^4^Wageningen University and Research, Greenhouse Horticulture, Wageningen, Netherlands

**Keywords:** hydroponics, biofortification, abiotic stress, induced resistance, nutrient management

## Introduction

Soilless culture can increase not only yield but also quality and safety of fresh produce and thus meet the demands of modern society. Soilless cultivation generally refers to any method of growing plants without soil as a rooting medium. The major advantage of soilless cultivation is the uncoupling plant growth from problems associated with soil, such as soilborne pests and diseases, non-arable soil, soil salinity, poor soil quality e.g., The increased interest in the commercial application of soilless cultivation in the last decades has encouraged intensive research activity focusing on the development of new growing systems and a better understanding of the crop physiology and its impact on quality aspects.

The cultivation on substrates is worldwide the primarily used soilless technique for fruiting vegetables and cut flowers. Water culture systems such as deep float techniques (DFT), nutrient film technique (NFT) and aeroponics— referred also as “hydroponics”— are often used for leafy vegetable production. The complete control of nutrition via the nutrient solution (NS) provides efficient tools for physiological and nutritional studies, improving product quality. The recycling and the control of the excess NS that drains off brings a considerable reduction in leaching of nutrients and plant protection products to the environment and saves water.

## Soilless Cultivation Through An Intensive Crop Production Scheme

One of the topics addressed in the current special issue on soilless culture is the nutrient needs and the quality characteristics of specialty vegetable crops when grown hydroponically, for which the available information in the scientific literature is scarce. In this context, Chatzigianni et al. evaluated two contrasting *Chichorium spinosum* ecotypes originated from a montane and a coastal-marine habitat and provided insights on their **salinity** tolerance and nutrient needs but also on the possibility to be used as promising germplasm resources for future breeding programs.

Salinity affects not only crop yield but also produce quality. To that direction, Chrysargyris, Tzionis et al. studied the *Tagetes patula* response to short-term exposure to moderate (50 mM) or high (100 mM) salinity. Results indicated that salinity decreased at one hand plant biomass but at the other the induction of non-enzymatic and enzymatic antioxidant mechanisms and short-term exposure to salinity and/or ethanol application during flower stage resulted in higher carotenoids and anthocyanins levels of flowers, which might be a new source of nutraceuticals.

In addition, Rouphael and Kyriacou reported **on salinity eustress** (positive stress) and **biofortification** as tools to improve product quality. Effective application of eustress, can elicit tailored plant responses involving activation of physiological and molecular mechanisms that can result in strategic accumulation of bioactive compounds necessary for adaptation to suboptimal environments. Chrysargyris, Michailidi et al. studied the effects of salinity on the physiological and biochemical responses of medicinal and aromatic plants focusing on lavender (*Lavandula angustifolia*). In that study, high (100 mM NaCl) salinity decreased plant growth, polyphenols, antioxidant capacity and essential oil yield, while low-moderate salinity levels maintained the volatile oil profile in lavender.

Ropokis et al. examined the **nutrient and water uptake** by *Capsicum annuum* as impacted by the cultivar and grafting and observed that different pepper cultivars may take up nutrients and water at different ratios under the same conditions. However, such variation was not found when pepper was grafted onto a *C. annuum* rootstock. They concluded that cultivar “Sondela” require higher Ca, Mg, and B concentrations than standard and higher K for cultivar “Bellisa.” In another paper dealing with the application of new technologies in hydroponics, Moon et al. studied the EC fluctuation of root-zone nutrient solutions in closed-loop soilless cultures using recurrent neural network (RNN), recording EC every 10 s for a period of 2.5 months in hydroponically grown sweet peppers (*C. annuum*). It was concluded that a single-layered algorithm showed the highest test accuracy, while deep learning algorithms could be applied with the addition of other environmental factors or plant growth.

In another paper exploring the possibilities of utilizing soilless culture to produce **functional food**, Asaduzzaman et al. investigated in four cultivars the production of low-potassium melon, by restricting potassium concentration in the supplied nutrient solution which resulted in production of melon fruits with 55–58% lower potassium content compared standard. Biofortification in soilless culture was the topic of a Incrocci et al., who studied iodine accumulation in hydroponically grown sweet basil (*Ocimum basilicum*). Different cultivars of sweet basil were screened with respect to their tolerance to iodine, since biofortification with iodine entails the use of tolerant cultivars to iodine, due to their ability to withstand higher concentrations of iodine in leaf tissues, rather than due to efficient exclusion of this element from the leaves.

Plant protection and chemical application in soilless culture is limited and alternative means of **disease control** are attracting research interest. Yin et al. assessed the effectiveness of essential oils of *Zingiber officinale* Roscoe as a biological pesticide toward root-rot diseases of a highly valuable medicinal herb (*Panax notoginseng*) when grown in a soilless cultivation system. The findings reveal that essential oils from plants might serve as promising sources of eco-friendly natural pesticides.

## Soilless Culture: Concluding remarks and future issues

The benefits of soilless systems, i.e., higher yields, high produce quality and the potential of control over emission of nutrients and plant protection products are greatly achieved in high-tech greenhouses which enable year-round production. In contrast, in low-tech greenhouses in countries characterized by a mild climate, the costs of soilless systems are not always recouped by higher yields because some other factor may limit production. Hence, in those countries soilless culture is mainly adopted when the problems originating from the soil become critical, water resources are limited, or the environmental pollution by nutrient leaching is serious (Savvas and Gruda, 2018). This is likely the main reason for the less extensive expansion of commercial soilless culture in most Mediterranean countries compared to north European countries.

Soilless culture is not merely a modern technology for greenhouse production of vegetables and ornamentals. The inherent feature of soilless culture to decouple cultivation of plants from the soil can be used in more sophisticated plant cultivation systems, such as vertical farming, in which plants are grown in multiple layers mounted in closed constructions using artificial lighting and full control of all climate parameter, which enable crop production on locations usually not suited for horticulture, like inner cities or deserts. The ultimate cropping systems are the bioregenerative life-support systems for production of fresh food to nourish astronauts in space colonies (Paradiso et al., 2014). Another application of hydroponics is aquaponics, which couples hydroponic production of vegetables or ornamentals with fish production, by utilizing fish excrement for crop nutrition (Tyson et al., 2011). Finally, soilless culture can be applied for urban agricultural production (Eigenbrod and Gruda, 2015).

## Author Contributions

NT prepared the outline of the manuscript and analyzed the special issue topics. DS wrote the concluding remarks and future issues. All authors wrote parts of the manuscript, improved the draft and revised the final version.

### Conflict of Interest

The authors declare that the research was conducted in the absence of any commercial or financial relationships that could be construed as a potential conflict of interest. The reviewer FD declared a past collaboration with one of the authors NT to the handling editor.
